# Characterization of Ovine A3Z1 Restriction Properties against Small Ruminant Lentiviruses (SRLVs)

**DOI:** 10.3390/v9110345

**Published:** 2017-11-17

**Authors:** Lorena de Pablo-Maiso, Idoia Glaria, Helena Crespo, Estanislao Nistal-Villán, Valgerdur Andrésdóttir, Damián de Andrés, Beatriz Amorena, Ramsés Reina

**Affiliations:** 1 Instituto de Agrobiotecnología, UPNA-CSIC-Gobierno de Navarra, 31192 Mutilva, Spain; lorena.depablo@unavarra.es (L.d.P.-M.); idoia.g@unavarra.es (I.G.); helena.crespo@unavarra.es (H.C.); ancad@unavarra.es (D.d.A.); bamorena@unavarra.es (B.A.); 2 Sección de Microbiología, Departamento de Ciencias Farmacéuticas y de la Salud, Facultad de Farmacia, Universidad San Pablo C.E.U. Ctra, Boadilla del Monte Km, 28003 Madrid, Spain; estanislao.nistalvillan@ceu.es; 3 Institute for Experimental Pathology, University of Iceland, Keldur, 112 Reykjavik, Iceland; valand@hi.is

**Keywords:** APOBEC3, small ruminant lentiviruses, restriction factors, deaminase domain, alternative splicing

## Abstract

Intrinsic factors of the innate immune system include the apolipoprotein B editing enzyme catalytic polypeptide-like 3 (APOBEC3) protein family. APOBEC3 inhibits replication of different virus families by cytosine deamination of viral DNA and a not fully characterized cytosine deamination-independent mechanism. Sheep are susceptible to small ruminant lentivirus (SRLVs) infection and contain three APOBEC3 genes encoding four proteins (A3Z1, Z2, Z3 and Z2-Z3) with yet not deeply described antiviral properties. Using sheep blood monocytes and in vitro-derived macrophages, we found that A3Z1 expression is associated with lower viral replication in this cellular type. A3Z1 transcripts may also contain spliced variants (A3Z1Tr) lacking the cytidine deaminase motif. A3Z1 exogenous expression in fully permissive fibroblast-like cells restricted SRLVs infection while A3Z1Tr allowed infection. A3Z1Tr was induced after SRLVs infection or stimulation of blood-derived macrophages with interferon gamma (IFN-γ). Interaction between truncated isoform and native A3Z1 protein was detected as well as incorporation of both proteins into virions. A3Z1 and A3Z1Tr interacted with SRLVs Vif, but this interaction was not associated with degradative properties. Similar A3Z1 truncated isoforms were also present in human and monkey cells suggesting a conserved alternative splicing regulation in primates. A3Z1-mediated retroviral restriction could be constrained by different means, including gene expression and specific alternative splicing regulation, leading to truncated protein isoforms lacking a cytidine-deaminase motif.

## 1. Introduction

Apolipoprotein B editing enzyme catalytic polypeptide-like 3 (APOBEC3; A3) proteins are cytidine deaminases capable of inhibiting replication of several retroviruses, DNA viruses, long terminal repeat (LTR) and non-LTR retroelements, and even eliminate experimentally transfected plasmid DNA [1]. The A3 family contains seven paralogs in humans (A3A-A3H) and is part of the activation-induced cytidine deaminase-AID/APOBEC gene family, which shares the characteristic zinc (Zn^2+^)-coordinating catalytic motif (His-X-Glu-X23-28-Pro-Cys-X2-4-Cys). Each A3 protein contains one or two Z-coordinating motif-containing domains, giving rise to a new classification based on the quantity and quality of the Z domains [2].

The complete picture of A3-mediated viral restriction mechanism is dependent on the targeted virus and not fully understood. Regarding lentiviruses, A3 main activity is to mutate nascent lentiviral cDNA after the clearance of RNA from the DNA/RNA hybrid. Such mutations generate highly deaminated non-functioning viral genomes that can be processed by the cellular DNA repairing system. A3 incorporated into virions (passenger A3) is the main cause of deamination evidenced by viral reverse transcripts displaying a high frequency of G-A mutations. However, A3G, A3F and A3A have also been shown to reduce virus infectivity in a cytosine-deaminase independent manner [3,4].

Several studies have found signatures of A3 editing activity on retroviral genomes clearly demonstrating that A3 proteins edit lentiviral DNA in vivo [5]. Among them, A3G and A3F have been the most studied due to both high antiviral activity in vitro and high expression levels in human T cells. A3A with a single Z1 motif has a negligible antiviral activity against human immunodeficiency virus (HIV-1) in vitro [6]. However, A3A is involved in the antiviral state of lentivirus target cells such as monocyte/macrophages or dendritic cells with proven antiviral activity in vivo [7].

The protein in small ruminants homologous to human A3A is called A3Z1. A3Z1 induction in ovine and caprine blood-derived macrophages is achieved upon treatment with interferon (IFN-γ) and has been proposed as a marker of the M1 profile of macrophage differentiation which is in turn, able to restrict HIV-1 and Small Ruminant Lentiviruses (SRLVs) replication at a post-entry step [8,9].

SRLV are present all over the world in sheep, goats and wild ungulates and cause a multisystemic disease exerting meningoencephalitis, arthritis, pneumonitis and/or mastitis [10,11]. SRLVs main target cells are from the monocyte/macrophage lineage but monocytes restrict viral replication until they differentiate into tissue macrophages, in which viral replication and protein expression are induced [12]. Besides the required *gag*, *pol* and *env* genes, SRLVs encode a series of accessory genes such as *vif, tat* or *vpr*-like and *rev* [13]. Among them, the accessory protein Vif has been extensively studied as the main A3 antagonist. HIV-1 Vif expression counteracts the hA3 antiviral effect by targeting the protein for degradation by the proteasome, preventing its incorporation into the virion [14,15,16]. SRLVs Vif can mediate degradation of sheep A3Z3 and A3Z3 orthologs in humans, macaques, cows and cats [17].

Sheep encode four functionally active A3 proteins (Z1, Z2, Z3 and Z2Z3) [18], whose cytosine deaminase enzymatic activity is not required for full levels of retrovirus restriction [19]. Artiodactyl A3Z2Z3 proteins, besides being fully resistant to HIV-1 Vif activity, have shown a broad antiviral restriction against HIV-1 and Murine Leukemia Virus (MLV) inhibiting their infectivity by 8 and 4-fold respectively [19].

In this study, we have explored A3 expression in the ovine monocyte to macrophage maturation process and its influence on SRLVs replication. A3Z1 downregulation (and not A3Z2, Z3 or Z2Z3) correlated with increased SRLVs viral replication in monocyte-derived and M2-polarized macrophages. In contrast, high A3Z1 expression levels correlate with SRLVs virus restriction in monocytes and M1-macrophages. Besides the full protein, additional A3Z1 truncated protein forms lacking the cytidine deaminase motif (A3Z1Tr) were detected following immune stimulation with IFN-γ, interleukin 4 (IL-4) or infection with SRLVs. Both proteins were efficiently incorporated into virions but restriction was only exerted by A3Z1, and was independent of viral Vif, despite protein-protein interaction.

## 2. Materials and Methods

This project has been approved by the local Ethics Committee for the use of animal samples from the University of Zaragoza (Government of Aragon), reference number PI15/14, project AGL2013-49137-C3-R (2014–2017). Requirements of the Spanish (RED53/2013) and the European Union (2010/63) animal protection policies were fulfilled.

### 2.1. Samples and Cells

Lung samples were collected in RNAlater buffer (Qiagen, Hilden, Germany) at necropsy from two sheep of the Rasa Aragonesa and Assaf breeds after euthanasia by intravenous injection of barbiturate overdose followed by exsanguination. For the caprine counterpart, peripheral blood mononuclear cells (PBMC) were isolated from ethylenediaminetetraacetic acid (EDTA)-blood by Lymphoprep gradient centrifugation (δ = 1.077; Asix-Shield, Oslo, Norway) from one Murciano-Granadina goat.

PBMCs from SRLVs-free Rasa Aragonesa sheep, checked by serology and PCR, were seeded in two wells at 10^6^ cells/well in 6-well plates and monocytes were isolated by adherence in RPMI complete medium (1% of vitamins, 10 mM sodium pyruvate, 1% non-essential amino acids, 1% l-glutamine, 50 µM β-mercaptoethanol, 1% antibiotics/antimycotics mix). One replica was kept in TRI Reagent^®^ (Invitrogen, Carlsbad, CA, USA) after three days of culture (monocytes) for further RNA extraction. Another replica was allowed to differentiate into blood-derived macrophages (BDM) for twelve days of culture in RPMI complete medium supplemented with 10% fetal bovine serum (FBS). BDM maturation using IFN-γ and IL-4, hallmark cytokines of the M1 and M2 profiles respectively, was also carried out as previously described [8]. BDMs were collected for RNA extraction and RT-PCR was carried out using specific primers for M1 (APOBEC3Z1, A3Z1) and M2 markers (Mannose Receptor, MR; and Specific Intercellular adhesion molecule-3-Grabbing Non-integrin, DC-SIGN), and for A3Z1 and A3Z1Tr quantification (Table 1).

Human embryonic kidney (HEK)-293T cell, T-immortalized goat embryo fibroblast cell line TIGEF (kindly provided by Dr. Yahia Chebloune), and ovine skin fibroblast OSF, were used for propagation of viral stocks and/or for restriction studies in vitro.

PBMCs from four human samples, isolated from a dialysis filter, and two from different Cynomolgus Monkeys were kindly provided by Dr. Sandra Hervás (CIMA, Pamplona, Spain).

### 2.2. RNA Extraction and cDNA Synthesis

Tissue samples (10 mg) were homogenized in a Micro-Dismembrator U using steel beads (Sartorius, Göttingen, Germany). Total RNA isolation from PBMC, BDM and lung samples was performed by chloroform extraction and isopropanol precipitation. RNA was treated with TurboDNaseI (Invitrogen, Carlsbad, CA, USA) and purified by extraction with phenol acid, chloroform, and ethanol precipitation. Total RNA (1 µg) was retrotranscribed to cDNA with Transcriptor First Strand cDNA Synthesis (Roche, Basel, Switzerland) using oligo-dT primers.

### 2.3. Viral Stocks

SRLVs viral stocks from the genotype A (strain EV1, Maedi Visna virus-MVV-like) [20] and from the genotype B (strain 496, Caprine Arthritis Encephalitis virus-CAEV-like) [21] both from ovine origin, were titrated on permissive skin fibroblasts in 96-well tissue culture plates by using the Reed-Muench method after 7 days of infection and used in in vitro infections as specified.

### 2.4. Amplification of Complete A3Z1/A3A and Sequence Analysis

Primers were designed outside the A3Z1 coding region targeting the 5′ and 3′-UTRs (A3Z1-Out-Fw and A3Z1-Out-Rv; Table 1) in order to amplify the complete ovine sequences. cDNAs were from lung samples of Rasa Aragonesa and Assaf sheep breeds and from PBMC of a Murciano-Granadina goat. PCR reactions were performed with the PhusionTM High Fidelity Polymerase (Thermo Scientific, Waltham, MA, USA); amplicons were cloned into Topo-Blunt plasmid (Invitrogen, Carlsbad, CA, USA) following manufacturer’s instructions and sequenced (Stab Vida, Caparica, Portugal).

Human and monkey A3A complete coding regions were amplified with PhusionTM High Fidelity Polymerase using primers based on GenBank sequences (A3A-Hs-Fw and A3A-Hs-Rv; Hs-Hommo sapiens. Table 1). Amplified fragments were cloned into pJET vector (Thermo Scientific) and sequenced.

Nucleotide and deduced amino acid sequences obtained were aligned with the ClustalX program (University College Dublin, Belfield, Dublin, Ireland). Protein domains, families and functional sites were deduced using ScanProsite tool (ExPASy, SIB Swiss Institute of Bioinformatics).

### 2.5. Quantification of Small Ruminant APOBEC3 Expression

Primer3 software (Version 4.1.0; Whitehead Institute for Biomedical Research, Cambridge Cambridge, MA, USA) [22] was applied to design primers (Table 1) to amplify each of the four A3 proteins (A3Z1, A3Z2, A3Z3 and A3Z2-Z3) nucleotide sequences described [18]. The additional A3Z1 spliceoform lacking the deaminase motif (A3Z1Tr) was detected with new forward primer (qA3Z1Tr-Fw3, Table 1) and maintaining the same reverse primer (qA3Z1-Rv). Quantitative RT-PCR was performed with cDNA from ovine samples using SYBR Premix Ex Taq (Takara, Tokyo, Japan). All samples were run in triplicate along with no template controls in an ABI Prism 7900 Sequence Detector (Applied Biosystems, Foster City, CA, USA). β-actin was used as housekeeping gene for relative quantification using the 2^−Δ*C*t^ or 2^−ΔΔ*C*t^ method. Obtained values were compared with non-parametric Mann-Whitney U Test (IBM SPSS Statistics for Windows, Version 23.0 IBM SPSS Statistics. Armonk, NY, USA, IBM Corp.) and also using REST software (Qiagen, Hilden, Germany).

Standard curves of each PCR reaction were constructed by amplifying 10-fold dilutions of plasmids containing each of the amplicons. The efficiency of each reaction was calculated as E = (10(−1/slope) − 1) × 100 and compared.

### 2.6. In Vitro A3Z1/A3Z1Tr Expression

Kozak consensus sequence (GCCACC) was included in the forward primer and two extra stop codons in the reverse (A3Z1-EcoRI-Fw and A3Z1-XhoI-Rv; Table 1) to amplify the complete ovine A3Z1 and the truncated isoform lacking deaminase motif (A3Z1Tr) using Assaf lung cDNA. The obtained fragments were sub-cloned into the EXN eukaryotic expression vector kindly provided by Greg Towers (University College London, London, UK) (pLNCX2 with an inserted HA-tag; Clontech, Mountain View, CA, USA) and sequenced. These constructions were used for transfection of TIGEF and 293T cells with 1 μg of plasmid per well using Jet Prime (Polyplus, Illkirch, France) at a ratio of 1:2 (μg DNA:μL Jet Prime reagent) according to the manufacturer’s instructions.

A3Z1 and A3Z1Tr mRNA detection in the transfected cells was carried out by full length RT-PCR and relative expression quantified by quantitative RT-PCR.

### 2.7. Viral Infection

IFN-γ, IL-4 or control stimulated BDMs were infected with SRLVs (MVV and CAEV-like) at 0.1 TCID50/cell. On day 7 post infection, supernatants were collected for retrotranscriptase activity (RT activity) quantification, performed with the HS-Lenti RT activity kit (Cavidi, Uppsala, Sweden) according to the manufacturer’s instructions. The test was repeated at least three times in all the experiments.

TIGEF transfected with A3Z1, A3Z1Tr and empty vector were infected with MVV-like SRLV at 0.5 TCID50/cell. In the interest of analyzing the activity of cellular A3Z1, clarified supernatants were used 48 h post-infection for RT activity determinations, as indicated above. In parallel to RT activity, virus titration of supernatants was also performed by Reed-Müench methodology.

HIV-1, MLV and Simian Immunodeficiency virus (SIV)-based pseudoviruses encoding green fluorescent protein (GFP) were prepared in 293-T cells transfected with A3Z1, A3Z1Tr, A3Z2Z3 or empty plasmid as described [16]. In addition, HIV-1 pseudoviruses carrying A3Z1 and A3Z2Z3 together with empty plasmid or A3Z1Tr were also prepared.

Cell culture supernatants containing HIV-1, MLV and SIV viral particles were harvested 2 days after transfection, purified by centrifugation in a Megafuge 1.0 (Heraeus, Hanau, Germany) centrifuge at 1800 rpm for 5 min and filtered through a 0.2-μm-pore-size membrane to remove any remaining producer cell. The purified supernatants were placed on naive 293T cells (dilution 1:2 pseudovirus) and the percentage of GFP positive cells was evaluated in a FACSCalibur (BD Biosciences, San Jose, CA, USA) after 48 h. All the experiments were performed in triplicate.

### 2.8. Immunoblot and Immunoprecipitation

293T cells were co-transfected using Jet Prime with 1 µg of A3Z1-HA (N-terminal) and increasing amounts of A3Z1Tr-HA (N-terminal) tagged proteins. Cell lysates were collected in Laemmli buffer (0.125 mM Tris (pH 7.5), 4% SDS, 4% glycerol, 1% 2-mercaptoethanol) and proteins were separated by SDS-PAGE (12% self-casted gels) and transferred onto nitrocellulose membranes (0.45 μm; Amersham Protan; GE Healthcare, Little Chalfont, UK). Membranes were blocked o/n at 4 °C or 1 h RT with PBS-T (20 mM Tris, 150 mM NaCl, 0.1% Tween 20) containing 5% non-fat dry milk. Anti-HA tag antibodies (rat monoclonal antibody, Roche, Basel, Switzerland) were diluted 1:500 in blocking buffer and incubated for 90 min. The chemiluminescence reaction (Super Signal West Dura, Thermo Scientific) was revealed in Gene Genius Bio Imaging System (G-BOX Chemi HR16, Syngene, Cambridge, UK).

A3 degradation by Vif was evaluated in 293-T cells co-transfected using Jet Prime with 1 µg of HA (N-terminal)-tagged A3Z1, A3Z1TR or A3Z2Z3 in EXN plasmid and 1 µg HA (C-terminal)-tagged Vif in pVR1012 plasmid. Cell lysates were harvested and immunoblotting was performed using anti-HA tag antibodies as described above.

For immunoprecipitation (IP), 293-T cells were co-transfected using Jet Prime with 1 µg of HA (N-terminal)-tagged A3Z1 EXN and FLAG (N-terminal)-tagged A3Z1Tr pN3 plasmids. After 48 h, lysates were collected and 10% of cell lysates were saved as an input control. Beads anti-FLAG (EZ view Red Anti-FLAG Affinity Gel, Sigma-Aldrich, Saint Louis, MO, USA) were washed 3 times (1 min each wash) with 500 μL of lysis buffer (Tris-HCl 50 mM, NaCl 500 mM, EDTA 1 mM, 2-mercaptoethanol 1 mM, Glycerol 5%, Triton 1% and NP40 1% at pH 7.5). Cell lysates were pre-cleared with beads conjugated to anti-FLAG antibodies and incubated for 16 h at 4 °C. After washing in lysis buffer, immunoprecipitated complexes were followed by SDS–polyacrylamide gel electrophoresis (PAGE) and immunostaining using anti-HA and anti-FLAG antibodies (mouse monoclonal anti-FLAG M2-Peroxidase antibody, Sigma-Aldrich, Saint Louis, MO, USA). Reverse immunoprecipitation using FLAG (N-terminal)-tagged A3Z1 pN3 and HA (N-terminal)-tagged A3Z1Tr EXN plasmids was also carried out. β-tubulin immunoblots were used as loading control.

Vif immunoprecipitation was evaluated on 293-T co-transfected using Jet Prime with 1 µg of FLAG (N-terminal)-tagged A3Z1 and A3Z1Tr pN3 plasmid and HA (C-terminal)-tagged Vif-pVR1012 plasmids. After 48 h, cell lysates were immunoprecipitated with beads conjugated to anti-FLAG antibodies (Sigma-Aldrich, Saint Louis, MO, USA) as described above. Reverse immunoprecipitation (IP) experiments using HA (N-terminal)-tagged A3Z1 and A3Z1Tr EXN and FLAG (N-terminal)-tagged Vif-pN3 plasmids were carried out.

### 2.9. Lentiviral Incorporation of A3Z1

Supernatants containing MVV-like and CAEV-like SRLVs obtained after a passage in TIGEF expressing A3Z1, A3Z1Tr and empty plasmid were harvested 2 days after infection, precleared by centrifugation in a Megafuge 1.0 (Heraeus, Hanau, Germany) centrifuge at 800× *g*, for 10 min and filtered through a 0.2-μm filter. Cell lysates were also obtained in Laemmli buffer. Virions were purified by gradient centrifugation through 20% sucrose for 5 h at 10,000× *g* in an SW32 Ti rotor (Beckman Coulter, Brea, CA, USA). Viral supernatants filtered and pelleted were resuspended in 100 μL of cold PBS. Protein content in purified virions was measured by Bradford assay (Biorad, Hercules, CA, USA) and A3Z1/A3Z1Tr incorporation into virus-like particles was checked. Immunoblots were developed with anti-HA antibodies.

HIV-1 vector expressing GFP was produced in human 293-T cells co-expressing HA-tagged A3Z1 and A3Z1Tr and supernatants were purified and probed as above.

SRLV packaging plasmid (pCAEV-AP) based on CAEV-Cork [23] (encoding alkaline phosphatase), kindly provided by Dr. Isidro Hötzel, was co-transfected in 293-T cells together with plasmids (pMDG) encoding envelope protein from vesicular stomatitis virus (VSV-G) as described [24]. 293-T cells were previously transfected with HA-tagged A3Z1, A3Z1Tr and empty plasmid resulting in the formation of CAEV based viral particles expressing AP pseudotyped with VSV-G protein. Pelleted viruses were purified and probed as above to check A3Z1 and A3Z1Tr incorporation.

### 2.10. Hypermutation

For hypermutation studies, SRLVs were produced in ovine skin fibroblast cells previously transfected with 1 µg of HA-(N-terminal) A3Z1, A3Z1Tr or empty vector per well using Jet Prime. The purified supernatants were placed on fresh permissive ovine skin cells. After 16 h, target cells were harvested and DNA was extracted using DNA Blood Mini Kit (E.Z.N.A, Omega Bio-tek, Norcross, GA, USA) and a region of the *gag-pol* genes was amplified (LTR-Fw-SP33; Table 1), cloned into pJET Cloning (Thermo, Waltham, MA, USA) and sequenced with pJET-Fw primer. Sequence analysis including *gag* region was conducted using Hypermutation 2.0 software (https://www.hiv.lanl.gov/content/sequence/HYPERMUT/hypermut.html, Los Alamos National Laboratory, Los Alamos, NM, USA).

## 3. Results

### 3.1. APOBEC3 Expression in Monocytes and Blood Monocyte-Derived Macrophages (BDM)

Since monocytes are less permissive than macrophages to SRLVs replication, mRNA relative expression of ovine A3 proteins (A3Z1, A3Z2, A3Z3 and A3Z2-Z3) was analyzed in these two cellular subsets by using quantitative RT-PCR. Efficiency values calculated for each standard curve ranged between 90% and 110%.

**Figure 1:**
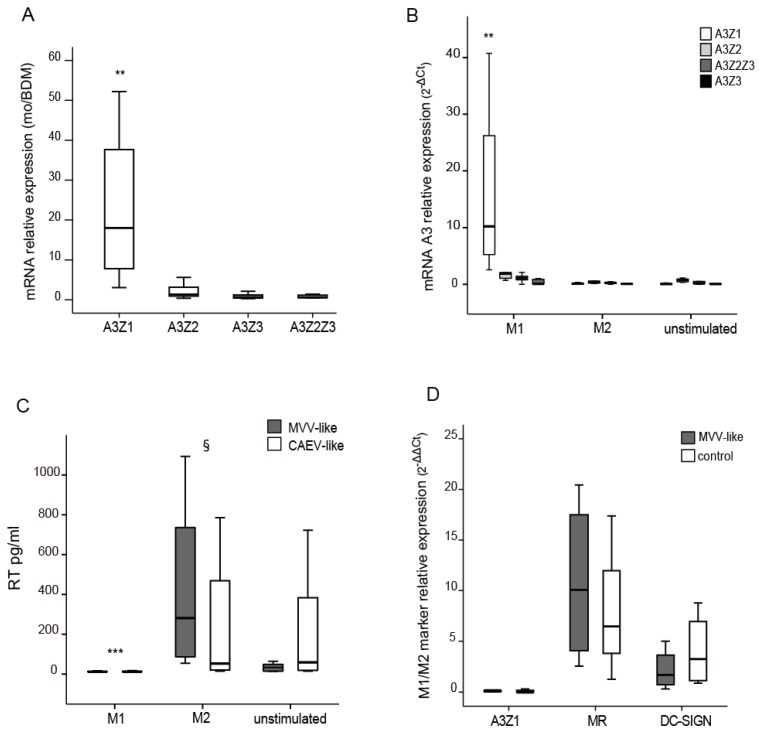
Relative Apolipoprotein B editing enzyme catalytic polypeptide-like 3 (APOBEC3) expression in Blood Derived Macrophages (BDM) and Small Ruminant Lentiviruses (SRLVs) replication. (**A**) A3Z1, A3Z2, A3Z3 and A3Z2-Z3 relative expression measured by quantitative RT-PCR in blood monocytes (mo) versus blood derived macrophages, BDM (2^−ΔΔ*C*t^); (**B**) A3Z1, A3Z2, A3Z3 and A3Z2-Z3 relative expression in BDM stimulated with interferon gamma, IFN-γ (M1), interleukin 4, IL-4 (M2) or control (unstimulated) for 6 days (2^−Δ*C*t^ × 100); (**C**) Retrotranscriptase (RT) activity (pg/mL) measured in clarified supernatants of stimulated BDM infected with 0.1 multiplicity of infection (MOI) of SRLVs strains from Maedi Visna virus (MVV)-like (grey bars) and Caprine Arthritis Encephalitis virus (CAEV)-like (empty bars) for 7 days; (**D**) A3Z1, MR, DC-SIGN relative expression in unstimulated BDM infected with SRLVs from MVV-like (0.1 MOI). Values are the median (±interquartile range) of at least three independent experiments, significantly lower (** *p* < 0.01; *** *p* < 0.001); significantly higher (^§^ *p* < 0.05) (paired Mann-Whitney U Test). (MR: Mannose Receptor. DC-SIGN: Specific Intercellular adhesion molecule-3-Grabbing Non-integrin).

APOBEC3 mRNA expression levels in monocytes and in blood-derived macrophages (BDM) were invariant in the case of A3Z2, A3Z3 and A3Z2-Z3. In contrast, A3Z1 mRNA expression levels were highly increased in monocytes (Figure 1A).

BDMs stimulated with hallmark cytokines of the M1 (IFN-γ) pattern of differentiation also showed significant differences in A3Z1 expression but not in A3Z2, A3Z3 or A3Z2Z3 (Figure 1B). IL-4 treatment did not induce A3 expression in BDM, showing values close to the control (*p* = 0.846), whereas IFN-γ stimulation strongly induced A3Z1 expression (*p* < 0.001). IFN-γ or IL-4-stimulated BDM showed completely different permissiveness to SRLVs replication. IL-4 and control stimulated cells showed high RT activity values for both MVV-like and CAEV-like strains assayed. However, viral RT activity in IFN-γ stimulated BDM was strongly inhibited (*p* < 0.001) (Figure 1C) along with an induction of A3Z1 expression (Figure 1B). MR and DC-SIGN were induced in the permissive M2 subpopulation confirming previous results [8]. A3Z1, MR and DC-SIGN expression did not modify after SRLV infection of macrophages (Figure 1D).

**Figure 2:**
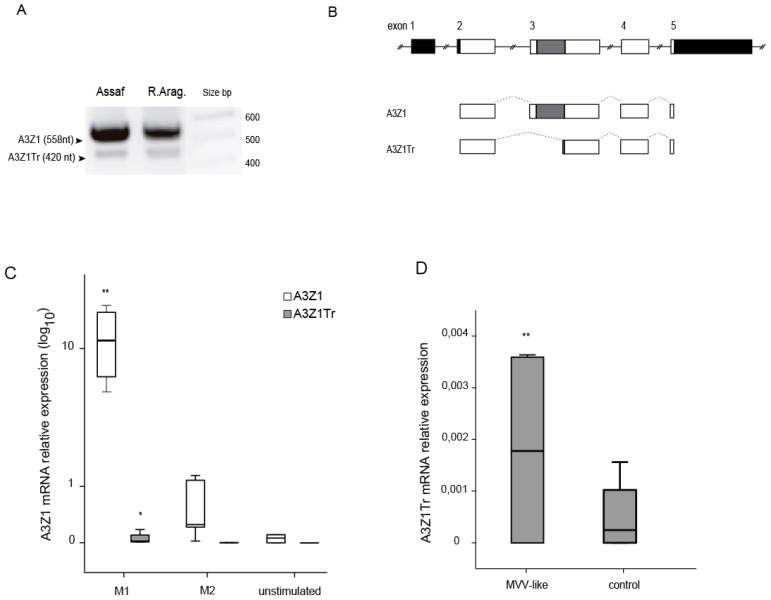
A3Z1 and A3Z1Tr detection. (**A**) Amplification of complete A3Z1 and the A3Z1Tr spliced variant from cDNAs samples of Assaf and Rasa Aragonesa sheep breeds; (**B**) ovine A3Z1 spliced variant diagram compared to the GenBank deposited sequence. Introns are represented by solid lines, exons by boxes and splicing sites by dotted lines. Untranslated regions, black; coding regions, white; cytidine deaminase motif, dark grey; frameshift, light grey; and dotted line boxes: no translated; (**C**) expression of A3Z1 and A3Z1Tr (2^−Δ*C*t^ × 100) in BDM treated with IFN-γ, IL-4 or pN3- control for 6 days; (**D**) relative expression of A3Z1Tr (2^−Δ*C*t^ × 100) in BDM infected with SRLVs from MVV-like 0.1 MOI. Values are the median (±interquartile range) of at least three independent experiments, significantly higher (* *p* < 0.05; ** *p* < 0.01) (paired Mann-Whitney U Test).

Besides the expected full sequence, other A3Z1 mRNA variants were also amplified and sequenced from the same RT-PCR reactions (GenBank: KM266653 to KM266660). Among them, a 420 nucleotide length amplicon was the most abundant and was present in all the samples used in the study (Figure 2A). This A3Z1 isoform presented an alternative splicing between nucleotides 141 and 280 of the coding sequence (amino acids 47 to 94, exon 3), corresponding to a shorter protein that lacks the entire cytidine deaminase domain and the zinc finger motif, hereafter named truncated A3Z1 (A3Z1Tr) (Figure 2B).

IFN-γ, IL-4 and control stimulated macrophages were analyzed for their A3Z1 and A3Z1Tr expression. As shown above, A3Z1 expression was higher in IFN-γ stimulated BDM compared with IL-4 and control BDMs. A3Z1Tr was significantly induced in IFN-γ and not in IL-4 or control BDMs (Figure 2C). SRLV infection also resulted in increased A3Z1Tr expression (Figure 2D) in contrast to A3Z1, which was not altered (Figure 1D).

### 3.2. A3Z1-A3Z1Tr Interaction

**Figure 3:**
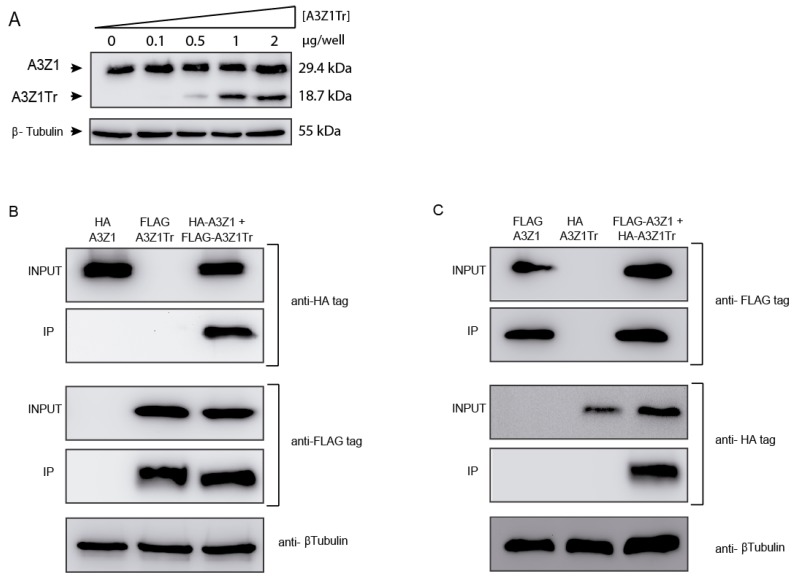
APOBEC3Z1 expression and interaction. (**A**) HA-tag detection in 293T cells transfected with 1 μg of A3Z1 and increasing amounts of A3Z1Tr; (**B**) 293T cells were co-transfected with plasmids expressing A3Z1 tagged with HA and A3Z1Tr tagged with FLAG epitopes and after 48 h were immunoprecipitated with anti-FLAG beads. Input lysates and co-immunoprecipitated samples (IP) were immunoblotted by anti-HA or anti-FLAG monoclonal antibodies; (**C**) reverse pull-down experiment. 293T cells were co-transfected with A3Z1-FLAG and A3Z1Tr-HA epitopes and after 48 h were immunoprecipitated with anti-FLAG beads. Input lysates and co-immunoprecipitated samples (IP) were immunoblotted by anti-HA or anti-FLAG monoclonal antibodies. β-tubulin blot was used as loading control.

Co-expression experiments were carried out in 293-T cells by transfection plasmids encoding A3Z1-HA and increasing quantities of A3Z1Tr-HA without altering protein expression (Figure 3A).

Using A3Z1Tr-FLAG protein and following anti-FLAG pull-down, anti-HA WB revealed the interaction between A3Z1 and A3Z1Tr (Figure 3B). Reverse pull-down experiments also showed interaction between the native and the truncated protein (Figure 3C).

### 3.3. Incorporation into Viral Particles

**Figure 4:**
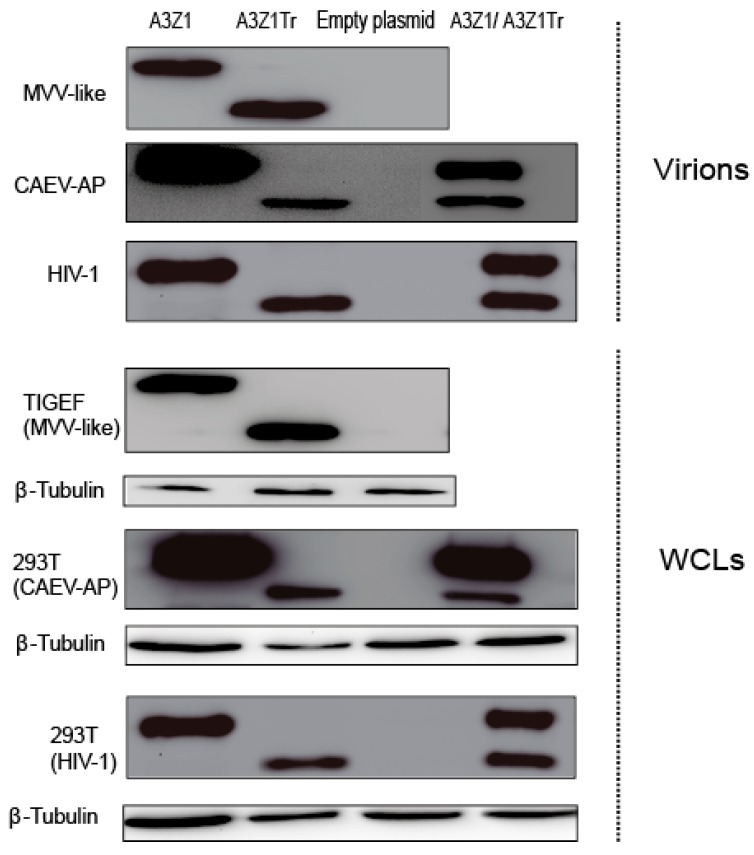
APOBEC3 encapsidation into SRLVs and HIV-1 virions. A3Z1, A3Z1Tr, empty plasmid and A3Z1/A3Z1Tr were transiently expressed in TIGEF and 293T cells, that were respectively infected with MVV-like SRLV or transduced with a CAEV-like pseudovirus (CAEV-AP) and a HIV-1 viral vector. Seven days post infection cleared supernatants were pelleted on sucrose gradient. Purified viruses and whole cell lysates (WCLs) and were blotted against HA. β-tubulin blot was used as loading control.

Since restriction may be dependent on deaminase activity in this is in turn dependent on A3 encapsidation into virions [25], we wanted to know whether A3Z1 could also be incorporated into virions. Transfected TIGEF and 293T cell lysates showed high amounts of A3Z1 and A3Z1Tr (Figure 4). Both HA-tagged proteins were evident in MVV-like purified viruses produced in infected TIGEF cells (Figure 4, upper panel) and also in CAEV-AP produced in 293T cells. Incorporation was also effective in co-transfected cells. A3Z1 and A3Z1Tr were also incorporated into HIV-1 particles very efficiently (Figure 4). Produced viral vectors were further used to transduce 293T cells in which no difference in infectivity was observed among A3Z1, A3Z1Tr or EXN containing viruses (*p* > 0.05 for all cases).

### 3.4. Vif Degradation

**Figure 5:**
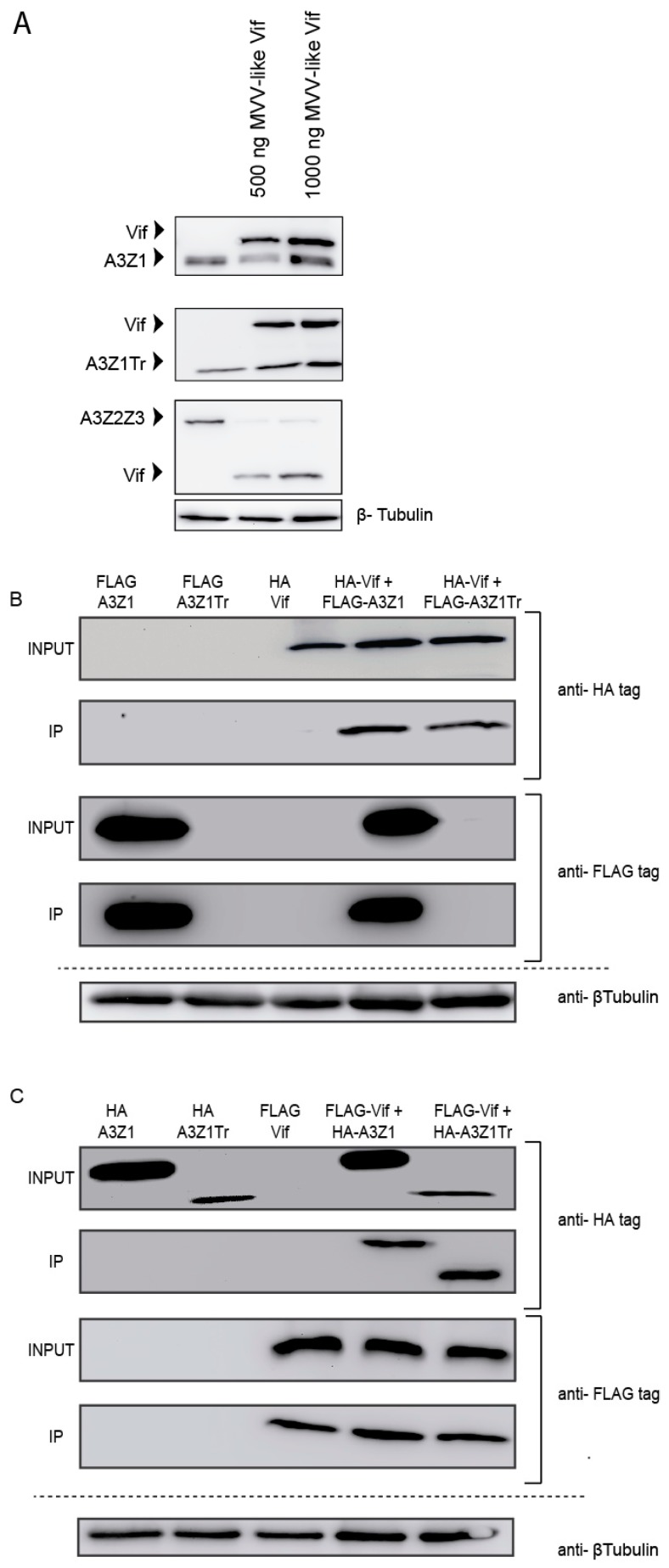
APOBEC3 resistance to MVV Vif. (**A**) HA-tag detection after 48 h in 293T cells co-transfected with 1 μg of plasmids expressing A3Z1 (upper panel), A3Z1Tr (middle panel) or A3Z2Z3 (down panel) with different concentrations of Vif plasmid; (**B**) 293T cells were co-transfected with plasmids expressing Vif-HA tagged and A3Z1Tr or A3Z1 tagged with FLAG epitopes and after 48 h were immunoprecipitated with anti-FLAG beads. Input lysates and co-immunoprecipitated samples (IP) were immunoblotted by anti-HA or anti-FLAG monoclonal antibodies; (**C**) reverse pull-down experiment, 293T cells were co-transfected with plasmids expressing Vif tagged with FLAG and A3Z1Tr or A3Z1 tagged with HA epitopes and after 48 h were immunoprecipitated with anti-FLAG beads. Input lysates and co-immunoprecipitated samples (IP) samples were immunoblotted by anti-HA or anti-FLAG monoclonal antibodies. β-tubulin blot was used as loading control. IP: Immunoprecipitation. INPUT: Input sample.

Despite efficient incorporation into virions, A3Z1 and A3Z1Tr were resistant to Vif degradation (Figure 5A, upper and middle panels) compared to A3Z2Z3 that was used as control (Figure 5A, lower panel). Interestingly, immunoprecipitation experiments showed Vif interaction with both A3Z1 and A3Z1Tr proteins (Figure 5B,C). Despite faint expression, A3Z1Tr-FLAG presence was confirmed in overexposed images and in WB in which the truncated protein was loaded alone, however, for the sake of a neat presentation of the results, overexposed images have been omitted.

### 3.5. APOBEC3Z1 Restriction of Retrovirus Infection

**Figure 6:**
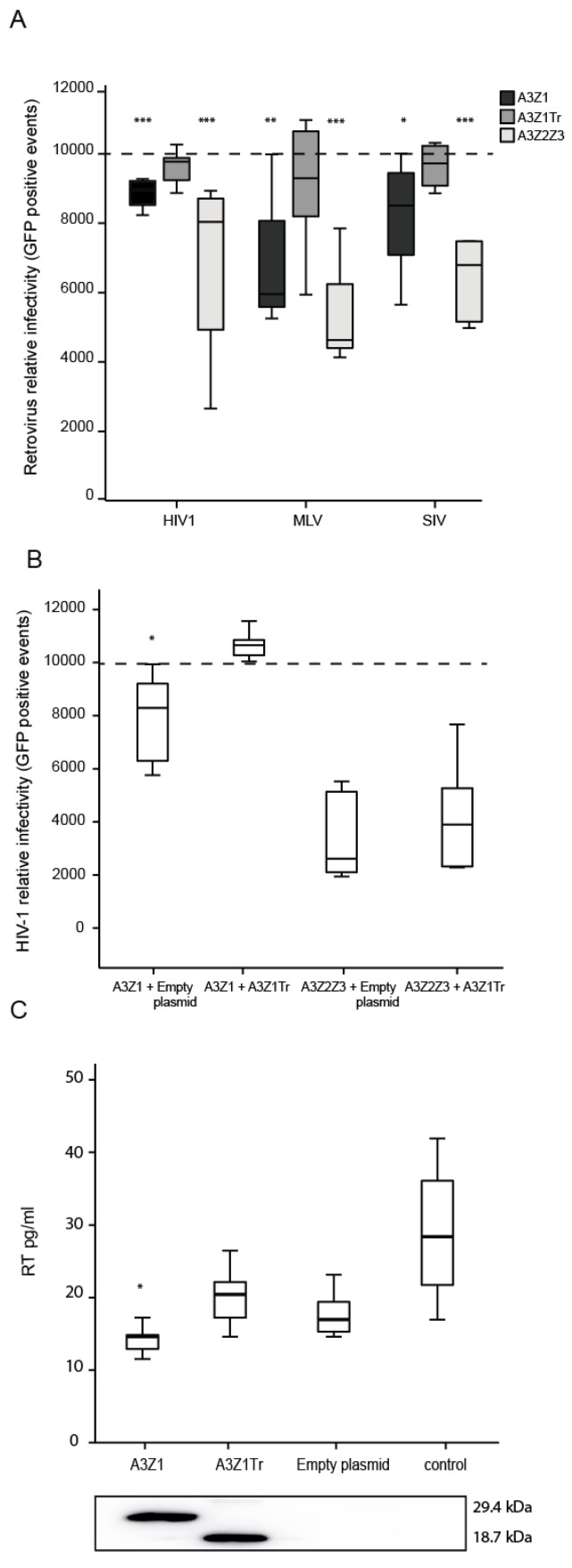
Retrovirus infectivity assay. (**A**) Relative infectivity of HIV-1, MLV and SIV-GFP pseudoviruses produced in the presence of A3Z1 (black bars), A3Z1Tr (dark grey bars) and A3Z2Z3 (light grey bars). Data are relative to the infectivity obtained with empty plasmid (discontinued line). Values are the median (±interquartile range) of at least three independent experiments, significantly lower (* *p* < 0.05; ** *p* < 0.01; *** *p* < 0.001) compared to empty plasmid; paired Mann-Whitney U Test); (**B**) relative infectivity of HIV-1 pseudoviruses produced in presence of A3Z1 and A3Z2Z3 supplemented with empty plasmid (control) or A3Z1Tr (spliced variant). Data are relative to the infectivity obtained with empty plasmid (discontinued line). Values are the median (±interquartile range) of at least three independent experiments, significantly lower (* *p* < 0.05) compared to empty plasmid; paired Mann-Whitney U Test); (**C**) RT activity values (pg/mL) upon MVV-like infection in TIGEF (0.5 MOI) expressing A3Z1, A3Z1Tr, empty plasmid and control cells (upper panel). HA-tag detection after 48 h post transfection (lower panel). Values are the median (±interquartile range) of at least three independent experiments, significantly lower (* *p* < 0.05) paired Mann-Whitney U Test.

Restriction was first evaluated in 293-T producer cells transfected with A3Z1 together with plasmids for the production of Vif-competent HIV-1, MLV or SIV GFP-encoding vectors. Retroviral produced particles transduced from 15 to 32% less 293-T cells when A3Z1 was incorporated. A3Z1Tr despite efficient incorporation into lentiviral particles did not inhibit the retroviral cycle. A3Z2Z3 induced the highest degree of retroviral inhibition in this cellular system with a 50% resistance against HIV-1 as previously described (Figure 6A) [26,27].

In the case of HIV-1, co-expression of A3Z1Tr and A3Z1 in the same virus producer cells significantly modified viral production since restriction was relieved. In contrast, A3Z1Tr expression did not modify the inhibition exerted by A3Z2Z3 (Figure 6B).

As in the human case [28], attempts to permanently express A3Z1 in SRLVs-permissive cells resulted in aberrant cells inappropriate for the restriction experiments. Antiviral properties of A3Z1 and A3Z1Tr were finally evaluated by transient transfection in TIGEF cells, in which HA-tagged A3Z1 and A3Z1Tr protein expression was detectable by Western blot (WB) (Figure 6C, lower panel). After infection with SRLVs, TIGEF-Z1 cells showed significantly lower RT activity values as compared to TIGEF-A3Z1Tr cells, TIGEF-empty plasmid cells and also compared to control cells (*p* < 0.05) (Figure 6C, upper panel). Titration of SRLVs in cell supernatants by the Reed-Muench method revealed that secreted virions were infective, resulting in viral titer values in line with RT activity results.

### 3.6. A3Z1 DNA Hypermutation Activity

**Figure 7:**
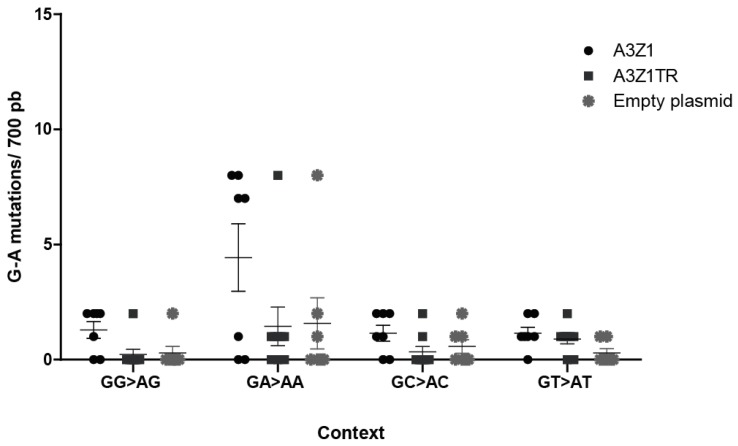
Nucleotide context of deamination. G to A mutations average in *gag* sequences obtained from ovine skin fibroblast (OSF) infected with SRLVs produced in OSF expressing A3Z1, A3Z1Tr or empty plasmid. Edited clones (A3Z1 *n* = 7, A3Z1Tr *n* = 9, Empty plasmid *n* = 7) in *gag* gene were aligned and analyzed with the Hypermut 2.0 program (https://www.hiv.lanl.gov/content/sequence/HYPERMUT/hypermut.html, Los Alamos National Laboratory, Los Alamos, NM, USA).

Permissive skin fibroblasts, with negligible endogenous A3Z1 expression, were infected with A3Z1 or A3Z1Tr containing SRLVs, and viral sequences obtained. Sequences spanning the *gag* gene indicated a high G to A mutation rate, in the GA→AA context, after infection by MVV-like containing A3Z1 (Figure 7). It can thus be concluded that the target sequence for ovine A3Z1 is GA-AA. Interestingly, G to A mutations in this context were also found in A3Z1Tr or empty plasmid transfected cells, as compared to sequences from untransfected cells, where no mutation was found in five clones.

### 3.7. A3Z1/A3A Sequence Analysis

Next, we studied the presence of A3Z1 and A3Z1 truncated isoforms in different primate species susceptible to lentivirus infection. A3Z1 sequences were successfully amplified from lung samples of the Rasa Aragonesa and Assaf sheep breeds and from PBMC of a Murciano-Granadina goat using primers located in the 5′ and 3′-UTRs. The 558 nucleotide coding sequence-CDS sequence analysis revealed a high similarity (0.971 for sheep and 0.967 for goat samples) between the obtained sequences and the one deposited in GenBank (GenBank: NM_001161379). These similarities were slightly lower when analyzing deduced amino acid sequences (0.930 and 0.935 for sheep and 0.908 for goat samples). Spanish sheep samples showed an amino acid deletion at position 134, whereas goat samples presented a different deletion at position 31, within a non-conserved region between sheep and goats. None of these deletions affected the cytidine deaminase motif, which was conserved region encompassing amino acids 57 to 95 (H-X-E-X23-28-P-C-X2-4-C). Both, sheep and goat sequences, also conserved a zinc finger C2H2-type domain between residues 88 and 108 overlapping with the cytidine deaminase motif. Kozak sequence context surrounding the A3Z1 M1 initiation codon was adequate [29].

Macaca fascicularis A3A sequences consisted of 609 nt and were quite divergent between the two individuals analyzed, with 19 variations in the nucleotide sequence and 11 amino acid changes. Truncated isoforms were also present specifically, an A3A 455 nt isoform that was only amplified from one of the samples (GenBank: KM266650 to KM266652).

**Figure 8:**
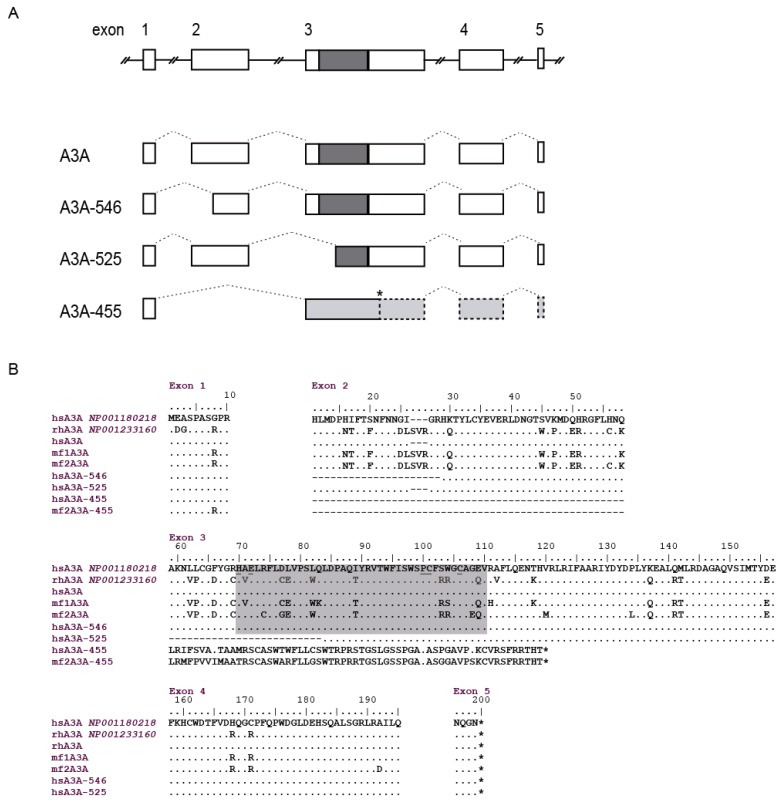
Human A3A transcripts. (**A**) Diagrams represent the different transcripts found in human A3A sequences obtained from PBMCs. Introns are represented by solid lines, exons by boxes and splicing sites by dotted lines. Coding regions, white; cytidine deaminase motif, dark grey; frameshift, light grey; and dotted line boxes: non-translated; (**B**) human A3A sequences obtained here from PBMC (hsA3A) were aligned with A3A sequences deposited in GenBank (with accession numbers) and A3A from *macaca fascicularis* (mf). Cytidine deaminase motif is highlighted in grey. Identical residues are identified by dots and absence of an amino acid by dashes. The numbering corresponds to that of the reference sequence.

Specific human A3A amplification, homologous to sheep A3Z1 protein, yielded non-specific amplicons when HTB-54, BEAS-2B, HEP-B2 or Caco-2 cultured cell lines were used as cDNA source. Successful amplification was achieved from four human PBMC and the obtained sequence was identical to the deposited in GenBank (GenBank: NM_001193289). Besides the deduced amino acid sequence with one cytidine deaminase domain between amino acids 70 to 110, shorter isoforms were also observed (Figure 8B) (GenBank: KM266646 to KM266649). Specifically, three different sized spliceoforms: (i) 546 nt, resulting in an 18 amino acid shorter protein with alternative splicing located between nucleotides 29 and 84 (amino acids 11 to 28, corresponding to exon 2), the cytidine deaminase domain remained intact and the methionine at position 13 was absent. (ii) 525 nt, lacking the cytidine deaminase domain due to an alternative splicing that produces a 25 amino acid deletion at position 59 to 83, corresponding to exon 3. (iii) 455 nt, with alternative splicing from nucleotides 29 to 175, which comprises the entire exon 2, that changes the frame at amino acid 11 encoding a 71 amino acid long non-characterized protein lacking also the cytidine deaminase domain.

## 4. Discussion

SRLVs replication occurs at low levels but at a constant rate leading to immune dysfunction and disease development in a process accomplished in years. However, in some cell types (i.e., monocytes) or under some circumstances (i.e., pregnancy), lentivirus replication can be naturally modulated [30,31,32] potentially providing clues for the designing of new therapeutic targets. Among proteins interfering the viral replication cycle, restriction factors of the innate immunity are becoming an attractive strategy to control infection spread. Specifically, tripartite motif-containing protein 5 (TRIM5α) and A3Z2Z3 have been recently described in small ruminants with promising results in restricting homologous as well as heterologous lentiviral infection [19,33].

In the present study, we explored ovine A3Z1 restriction properties against homologous viruses (SRLVs) in the context of macrophage maturation depicting the mechanisms involved and investigating Vif involvement as well as restriction against heterologous viruses (HIV-1). In addition to full-length A3Z1, transcriptional analysis revealed the presence of additional spliceoforms lacking the entire cytidine deaminase motif (A3Z1Tr) indicating that A3Z1 isoforms lacking the deaminase motif are naturally present.

SRLVs replication is induced when maturation from monocyte into macrophages takes place [12,34] presumably due to the induction of transcription factors needed for viral protein expression. Additionally, as shown here, A3Z1 expression was dramatically reduced in BDM as compared with parental monocytes likely contributing to viral restriction observed in the latter. In line with our results, decreased A3A expression during monocyte to macrophage differentiation has been associated with resistance to HIV-1 [7,28], indicating the presence of a conserved restriction mechanism among mammals susceptible to lentivirus infection.

Ovine M1-macrophages obtained upon stimulation with IFN-γ were resistant to SRLVs infection and presented high levels of A3Z1 mRNA (this study and [8]), whereas M2 susceptible to SRLVs infection, showed low A3Z1 levels, positioning A3Z1 in the SRLVs (MVV and CAEV-like) restriction pathway. A3Z1Tr was also induced upon BDM stimulation with cytokines or SRLVs infection suggesting a role in immune activation.

Viral restriction was cell type dependent since A3Z1-expressing TIGEF, with similar A3Z1 mRNA levels to M1 macrophages, faintly restricted the replication of SRLVs Vif-competent strains. Similarly, A3A restriction of HIV has been demonstrated in myeloid but not in cells from other lineages [7,28,35,36].

In addition, although A3Z1 restriction compared to A3Z1Tr in vitro was likely dependent on the deaminase activity, since deaminase motif was not present in A3Z1Tr, hypermutation was also found in viruses obtained after infection of cells expressing A3Z1Tr or the empty plasmid. In this regard, human A3A is induced by interferon following administration of foreign DNA by transfection [37]. Whether the induction of endogenous A3Z1 proteins in TIGEF transfected with plasmids is covering up real A3-derived effect is now under investigation.

Little information limited to humans is available regarding A3 splicing variants and their influence on restriction. A3G truncated forms, which are expressed at much lower levels than native protein, do not interact with Vif and lack antiviral activity [38]. A3H splicing variants have different antiviral activities depending on protein expression [39]. A3F splicing variants despite being highly expressed and conserving cytidine deaminase activity, differ in their sensitivity to viral Vif and are incorporated into viral particles less efficiently than full protein, thereby displaying decreased antiviral activity [38]. Alternatively, spliced truncated isoforms may be a mechanism to reduce the amount of full-length proteins [40]. Here we have determined different splicing rates of A3Z1 depending on the stimulus on macrophages, being immunological stimulation of BDM with IFN-γ and more significantly SRLVs infection, inducers of the spliced protein. This was also found in THP-1 PMA-derived macrophages in which A3ATr expression was induced upon transduction with different retroviral vectors such as HIV-1 or MLV-B/N.

Efficient co-expression in 293T cells enabled interaction experiments demonstrating co-immunoprecipitation of A3Z1 and A3Z1Tr. This interaction could alter full-length protein function suggesting a possible regulation mechanism consisting in binding and negative dominance, as described in the case of MYD88s [41]. Indeed, A3Z1Tr relieved the restriction observed against HIV-1 when co-expressed with A3Z1, but did not alter the restriction exerted by the divergent A3Z2Z3. However, the presence of A3Z1Tr did not influence the SRLVs restriction observed in M1-macrophages obtained upon IFN-γ induction.

A3Z1 lentiviral restriction was likely independent on the presence of viral Vif since transduction (HIV-1) and infection (SRLVs) with Vif-competent viruses was affected. This could argue for the presence of extra elements needed for the Vif-APOBEC3Z1 interaction, which were not present in the culture conditions used here [42]. Transcription factor CBFβ, as well as viral RNA, is necessary to form the bridge between primate A3 proteins and Vif [26]. Recently, SRLVs Vif has been described to follow alternative mechanisms to degrade A3 using CypA as cofactor [26,27]. Whether A3Z1 follows the same pathway in Vif degradation co-expression experiments is unknown. On the other hand, significant amounts of A3G have been detected in Vif-competent HIV virions produced in 293T cells, clearly showing that under certain circumstances, Vif does not lead to complete depletion of A3 [16,43]. Interestingly, and in contrast to A3A [28] both A3Z1 and A3Z1Tr were efficiently incorporated into SRLVs (MVV and CAEV-like) and HIV viral particles bypassing Vif-mediated degradation.

The long interaction between lentiviruses and their hosts has led to the idea that inter-species A3, evolved under the pressure of a divergent Vif, could be good candidates to face human lentiviruses. Following this model, ovine A3Z2-Z3 and A3Z3 proteins are able to restrict HIV-1 irrespective of HIV-Vif expression by deaminase dependent and independent pathways. Interestingly, Vif from SRLVs is able to efficiently degrade human, macaque, cow and cat’s A3Z3 [17,19]. In this study, A3Z1 restricted HIV-1 in 293-T producer cells (Figure 6A). However, experiments have not been conducted in myeloid cells which could be the proper cell type to test A3Z1/A3A restriction profile.

The overall A3A deaminase activity in vivo is likely determined by a complex balance between stimulatory and inhibitory pathways, truncated isoforms being one of such potential elements assuring viral defense while minimizing host DNA deleterious effects [44]. Increased functional diversity may have implications in expression regulation, antiviral activities, Vif resistance, etc., all of them influencing the design of new therapeutic measures as well as in the development of tumorigenic processes in which cytidine deaminases are relevant.

## Figures and Tables

**Table 1:** Primer pairs used.

Name	Sequence	Localization	Amplicon Size (nt)	Reference
A3Z1-Out-Fw	AGGACTCGGAGCCAGGGACGA	Z1 3′-UTR	621	This work
A3Z1-Out-Rv	TCCTGCCATCTTAGAGAGGCTG	Z1 5′-UTR		
A3A-Hs-Fw	ATGGAAGCCAGCCCAGCATC	A3A met	600	This work
A3A-Hs-Rv	TCAGTTTCCCTGATTCTGGAG	A3A stop		
qA3Z1-Fw	TCCGTTCTTGGAATCTGGAC	Z1 CDS	151	[8]
qA3Z1-Rv	GTATAGATGCGGGAGGCAAA	Z1 CDS		
qA3Z2-Fw	TTGAACCACCCTGTCTTTCC	Z2 3′-UTR	188	This work
qA3Z2-Rv	CAGGCTTCAGGGTTGTTGTT	Z2 3′-UTR		
qA3Z3-Fw	GGGCGAGGAGATTGTGTTT	Z3 CDS	182	This work
qA3Z3-Rv	AAGCAGCCTTTGTCAAGCAT	Z3 CDS		
qA3Z2-Z3-Fw	CAGGCCTTGGAAGAAACTGA	Z2 CDS	151	This work
qA3Z2-Z3-Rv	CCTCCGGTAGTAAGGTGGTG	Z3 CDS		
qA3Z1Tr-Fw3	GCTTTGTGCGCAACAAGAAA	Z1 CDS	85	This work
MR qPCR-Fw	TGGCAAATCCAGTTGTTAAGATGTT	MR CDS		
MR qPCR-Rv	AGAATGTTGAATACTGTGGCGAGTT	MR CDS	91	[8]
DC-SIGN-Fw	GGTTCCGGAGTCTGACTGAAGTT	DC CDS		
DC-SIGN-Rv	GGTCAGGCGCTGTAGGATCTC	DC CDS	73	[8]
β-actin-Fw	CTCACGGAGCGTGGCTACA	Actin CDS		
β-actin-Rv	GCCATCTCCTGCTCGAAGTC	Actin CDS	88	[8]
A3Z1-EcoRI-Fw	TTTGAATTCGCCACCATGGATGAAAACACCTTCACTG	Z1 met	588	This work
A3Z1-XhoI-Rv	TTTCTCGAGCTACTATCAGTTTTGCTGAGCCCTGA	Z1 stop		
LTR-Fw	TGACACAGCAAATGTAACCGCAA	LTR	5126	This work
SP33-Rv	CTTCCCCTTCCCAGAGTACCTGAG	POL		

CDS: Coding DNA sequence; UTR: Untranslated region; Sequences are listed 5′ to 3′; Restriction enzyme recognition sites are underlined. Hs: Hommo sapiens. MR: Mannose Receptor. DC-SIGN: Specific Intercellular adhesion molecule-3-Grabbing Non-integrin. LTR: long terminal repeat. POL: SRLV *pol* gene.
